# Perinatal outcomes in 35 children with cartilage hair hypoplasia

**DOI:** 10.1186/s13023-026-04364-9

**Published:** 2026-04-30

**Authors:** Ellamaija Kasanen, Sanna Toiviainen-Salo, Svetlana Vakkilainen, Leena Kainulainen, Laura Tanner, Outi Makitie

**Affiliations:** 1 Research Program for Clinical and Molecular Metabolism, Faculty of Medicine, University of Helsinki, Helsinki, Finland; 2 Department of Pediatric Radiology, HUS Medical Imaging Center, Helsinki University Hospital, Helsinki, Finland; 3 Children’s Hospital and Pediatric Research Center, University of Helsinki and Helsinki University Hospital, Helsinki, Finland; 4 Department of Pediatrics and Adolescents, Turku University Hospital, University of Turku, Turku, Finland; 5 HUSLAB Department of Clinical Genetics, Helsinki University Hospital, Helsinki, Finland; 6 Department of Medical and Clinical Genetics, University of Helsinki, Helsinki, Finland; 7 Department of Molecular Medicine and Surgery, Karolinska Institutet, and Clinical Genetics, Karolinska University Hospital, Stockholm, Sweden; 8 Folkhälsan Research Center, Helsinki, Finland

**Keywords:** Cartilage-hair hypoplasia, Prenatal diagnosis, Prenatal ultrasonography, Newborn, Delivery, Skeletal dysplasia

## Abstract

**Background:**

Cartilage-hair hypoplasia (CHH), an autosomal recessive skeletal dysplasia due to RMRP mutations, is characterized by short stature, immunodeficiency, anemia, and increased malignancies. Growth failure has its onset prenatally. Birth and neonatal care in pregnancies with fetal skeletal dysplasia have been studied in some other skeletal dysplasias but there have been no previous studies on these aspects in CHH. In this retrospective cohort study, we reviewed patient records for 35 Finnish children with CHH born in 2000–2023 to evaluate the course and management of pregnancies and deliveries and the prenatal and neonatal findings.

**Results:**

Abnormal growth was observed in prenatal ultrasound in 26/30 (87%) children. Shortness was reported especially in the humeri and femora, but the ultrasound findings also included other abnormalities such as a small rib cage. Abnormal ultrasonographic findings mostly visualized during the second trimester. A prenatal genetic diagnosis was made in 6/34 (18%) cases. The median age at time of postnatal genetic diagnosis was 1 month. The Finnish founder mutation was observed in 94% of all cases. Most children (27/33; 82%) were born full-term but 6 children (18%) were born preterm (at 30 + 4–36+4 weeks). Less than half (11/24; 46%) were born by vaginal delivery and the others by elective (5/24; 21%), urgent (4/24; 17%), or emergency Csection (4/24; 17%). Breech position and other malpresentations were more common (30%) than in the general population (3–4%) and accounted for 10/18 (56%) of the Csection indications. The average 1 min Apgar score was 7.4. The median birth length for full-term neonates was 44.5 cm (40.0–50.0 cm) for boys (*n* = 10) and 44.0 cm (37.0- 48.0 cm) for girls (*n* = 16). The median birth length Z-score, adjusted for gestational age, for all was −3.9 (−7.5- −1.0) and below −2.0 in 84%. In 7/19 (37%) cases respiratory support was needed postnatally.

**Conclusion:**

In conclusion, in most CHH pregnancies, growth failure was detected prenatally, the pregnancies were carried to full-term but less than half were born by normal vaginal delivery. At birth 16% had normal length. Respiratory challenges accounted for the majority of the neonatal complications.

## Introduction

Cartilage-hair hypoplasia (CHH) (OMIM #250250) is an autosomal recessive skeletal dysplasia caused by pathogenic variants in the *RMRP* gene. Although CHH is globally rare it is enriched in the Amish and Finnish populations, and the incidence in Finland is reported to be 1:23,000 live births [1]. The Finnish founder variant (FFV) n.72A > G (also known as n.71A > G and n.70A > G in previous literature) explains 92% of the cases found in Finland [2]. CHH is characterized by disproportionate short stature; average adult heights are 131.1 cm and 122.5 cm for males and females, respectively [3]. Extraskeletal manifestations may include combined immunodeficiency with increased susceptibility to infections and pulmonary complications [4–9].

Patients with CHH have abnormal erythropoiesis and may present with severe anemia in infancy [8, 10]. Similarly, the incidence of Hirschsprung disease (HD) is high and the presence of HD has been associated with an overall severe phenotype and a poor prognosis in the 20^th^, but not in the 21^st^, century [11–14]. CHH patients are at an increased risk for malignancies, especially basal cell carcinoma and non-Hodgkin lymphoma [15, 16]. These complications contribute to the elevated mortality associated with CHH [16]. While there is currently no targeted treatment for chondrodysplasia in CHH, the quality of life and management of comorbidities can be improved with timely diagnosis. Heterozygous carriers of *RMRP* variants do not present with CHH manifestations and have no increased risk of malignancies [1, 6, 17, 18].

The suspicion of CHH may arise during the prenatal period triggered by the intrauterine growth disturbance involving especially the long bones [19–21]. Prenatal evaluation by a specialist is recommended for all pregnancies with suspected fetal skeletal disease [22]. Prenatal diagnostics of CHH has been available since 1990s, first through DNA markers [23]. After the discovery of the gene in 2001, a more targeted approach has been applied, although it has rarely been used in pregnancies without previous family history of CHH [2, 19–21, 23, 24].

Very limited data are available on perinatal outcomes in CHH. Birth lengths are reported to be below average. In an older study on the Finnish cohort, a birth length below −2.0 SD was reported in 70% of the patients [12]. On the other hand, in a Japanese cohort with genetic backgrounds that differ from the Finnish population, none of the 6 patients had a birth length below −2.0 SD [25]. Shorter birth length, decreased T-cell production and function have all been reported to be important factors in predicting severe infections in CHH [11]. It has been hypothesized that birth length can reflect the degree of cell proliferation defect in *RMRP* deficiency [11]. Therefore, the genotype as well as the perinatal characteristics including birth length are likely to be important for the management and prognosis of neonates with CHH.

The data on prenatal and neonatal outcomes, beyond birth length, in CHH are very limited and there are no previous studies describing perinatal aspects of children with CHH. This study evaluated the prenatal findings and the course of pregnancies and deliveries in newborns with CHH in the Finnish population.

## Cohort & methodology

### Research permits

This study is part of our research program on clinical, genetic and epidemiological aspects of skeletal dysplasias in Finland. An ethical approval was obtained from the Research Ethics Committee of the Hospital District of Helsinki and Uusimaa (HUS/836/2018 and HUS/564/2024). Data were collected from patient records, and therefore no patient consent was required according to Finnish laws.

### Patient cohort

For the present study we reviewed data for 35 children with CHH. All the children were included in the Finnish Skeletal Dysplasia register and born between January 1^st^ 2000 and December 31^st^ 2023. The children were born at university hospitals or regional hospitals in different parts of Finland. All Finnish children with CHH are followed at university hospitals as recommended and most children visit Helsinki and/or Turku University Hospitals for specialist care [26]. Data on diagnosis, family history, pregnancy, delivery, and neonatal period were gathered from the patient records. The birth lengths were corrected for gestational age [27]. In case the exact day on the gestational week was unavailable we used the first day of the gestational week.

### Prenatal care in Finland

In Finland all pregnant people are offered prenatal screening free of charge. The screening program consists of 1^st^ and 2^nd^ trimester screenings. The 1^st^ trimester combined screening takes place between 10^+1^ and 13^+6^ weeks and contains a general ultrasound examination and measurement of nuchal translucency (NT) in addition to screening for most common trisomies. The 2^nd^ trimester morphology ultrasound is performed between 18^+0^ and 21^+6^ gestational weeks.

Further evaluation by a specialist is offered if the screening or examination results are abnormal or if there is a known risk for a genetic disorder because of positive family history or confirmed carriership of an early-onset genetic disease in a parent.

### Genetic testing

The *RMRP* mutation analyses were performed from DNA isolated from amniotic fluid or chorionic villus prenatally or from peripheral blood postnatally by Sanger sequencing at Laboratory HUSLAB, Finland, or as part of a research project at Folkhälsan Institute of Genetics, Helsinki (only postnatal samples) [2, 11, 28]. Primers for the *RMRP* (GRCh37/hg19) were designed using Primer3 V.0.4.0 for the gene, with a minimum of 60 bases of flanking regions adjacent to the coding region [28]. Genomic reference NG_017041.1 and RNA reference sequence NR_003051.3 were used in analyses with Sequencer V.5.0 (Gene Codes, Ann Arbor Michigan, USA) [28].

## Results

### Genetics and family history

The study cohort included 35 children with a diagnosis of CHH. All children were born between 2000 and 2023. All 35 children had a genetically confirmed diagnosis of CHH. Genetic variant data was available for all children, 33 of whom (94%) tested positive for the FFV n.72A > G (rs199476103). Most of the children (28/35) were homozygous for the FFV whereas 5/35 were compound heterozygotes for FFV and n.264G > T (rs727502774) (previously marked n.263G > T). In the remaining two cases where FFV was absent the genotypes were n.-28_-7 duplication (rs1823640030) combined with n.264G > T, and n.-22_-13 duplication (rs1554651507) combined with n.264G > T. Family history was available for 28 children of which eight (29%) had 1–2 older siblings with CHH. None of the CHH patients’ parents had been diagnosed with CHH. Clinical characteristics of the cohort are presented in Table 1.

**Table 1:** Characteristics of the cohort of 35 Finnish subjects with cartilage-hair hypoplasia

Characteristics	All patients *n* = 35	
Sex		
Male	17/35	(49%)
Female	18/35	(51%)
Genotype	available *n* = 35	
* RMRP* n.72A > G/n.72A > G	28/35	(80%)
* RMRP* n.72A > G/n.246G > T	5/35	(14%)
other	2/35	(6%)
Timing of diagnosis	available *n* = 34	
Prenatal	6/34	(17%)
Postnatal	28/34	(82%)
Gestational week at birth	available *n* = 33	
≤h34+0	3/33	(9%)
h34+1 - h36+6	3/33	(9%)
≥h37+0	27/33	(82%)
Birth length†	available *n* = 32	
below −4.0 SD	12/32‡	(38%)
from −4.0 to −2.0 SD	15/32§	(46%)
from −2.0 to +2.0 SD	5/32¶	(16%)

† Birth lengths were adjusted for gestational age and sex [27]

‡ Including 4 preterm neonates

§ Including 1 preterm neonate

¶ Including 1 preterm neonate

The fact that the *RMRP* gene is noncoding RNA transcript poses challenges to the clinical classification of *RMRP* variants. Especially the absence of a translated protein combined with the scarcity of functional in vitro studies and variability in phenotype make the classification of *RMRP* variants according to the ACMG guidelines difficult. The ClinGen Criteria Specification Registry has published an interpretation guide for the *RMRP* gene [29]. Data on the classification of the variants detected in the cohort are presented in Table 2.

**Table 2:** Classification of *RMRP* variants in the cohort of 35 children with cartilage-hair hypoplasia

Reference sequence	Variant	dbSNP	Clinvar ID	ACMG Classification	ACMG Criteria	Other names for the variant	Additional information
NR_003051.4	n.72A > G	rs199476103	14208	Pathogenic	PM3 (VS), PM2, PS3, PP1, PP4	NR_003051.3:n.71A > G, previously g.70A > G	Finnish founder variant [24]
NR_003051.4	n.246G > T	rs727502774	14209	Pathogenic	PP1, PP4, PM2, PM3 (VS)	NR_003051.3:n.263 G > T	Severe Combined Immunodeficiency Disease VCEP curated variant
NC_000009.12	n.-28-7dup	rs1823640030	1454467	Pathogenic	PM1 (S), PM2, PM3, PP4	NC_000009.12:g.35658026_35658047dup	
NR_003051.3	n.-22-13dup	rs1554651507	14210	Pathogenic/Likely pathogenic	PM1 (S), PM2, PM3 (VS), PP1, PP4	NC_000009.12:g.35658031_35658040dup	

Note: ACMG classification criteria are not directly applicable as *RMRP* is not a protein-coding gene. Specific guidelines for variant interpretation have been suggested [29]. VS = very strong, S = strong

### Ultrasonography and timing of diagnoses

Data about prenatal ultrasonography findings were available for 30 children. In 26/30 (87%) cases, shortened long bones and/or abnormal growth were observed during pregnancy. Shortness was observed especially in the humeri and femora. Curving of long bones was not reported. Findings also included a small or narrow rib cage, hypoplastic lungs, enlarged segments of the bowel and, in one case, a disproportionately large head. No increased NT or signs of fractures were reported prenatally. The ultrasonographic findings are presented in Table 3.

**Table 3:** Ultrasound findings of the cohort of 35 Finnish subjects with cartilage-hair hypoplasia. IUGR: intrauterine growth restriction

Ultrasound findings	Data available *n* = 30
Short long bones	25
Small ribcage or hypoplastic lungs	5
No abnormalities reported	4
IUGR	1
Oligohydramnios	1
Large head	1
Enlarged segments of the bowel	1

There were limited data available on the specific timing of the primary abnormal ultrasonographic findings, however, we were able to determine the time point in seven cases. In 2/7 (29%) cases the abnormalities were visualized at the first trimester ultrasound and in 4/7 (57%) cases, at the second trimester ultrasound. In 1/7 (14%) cases the first abnormal findings were observed at 26 gestational weeks in an additional ultrasonographic examination performed at the request of the parents.

In 4/30 cases, no abnormal ultrasound findings were reported prenatally. However, all of these four children were born shorter than average, ranging from −4.5 SD to −1.0 SD. Genetically, two of these children were compound heterozygotes (one child: n.72A > G combined with n.264G > T; the other child n.264G > T combined with a rare n.-22_-13 duplication). The remaining two were homozygous for the FFV. There were no reports of incomplete participation in scheduled prenatal screenings but based on the limited data we were not able to confirm whether the parents took part in the ultrasound examinations, the growth delay had been missed or it had only become apparent after the second trimester morphology examination.

Data about the timing of diagnosis was available for 34/35 children. In 6/34 (18%) cases, a genetic diagnosis was reached prenatally triggered by the presence of short long bones and a fetal phenotype matching CHH. There was an older sibling with CHH in one case and in the five remaining cases there was no family history of CHH. For prenatal genetic diagnosis either chorionic villus sampling or amniocentesis was performed. Information on the gestational week at the time of prenatal diagnosis was not available. In 28/34 (82%) cases, the diagnosis was made postnatally. The age at time of postnatal diagnosis was available for 18 children, with the median age being 1 month (range 0–20 months).

There were various reasons for the small number of definite prenatal genetic diagnoses in the cohort. In several cases, the abnormalities were not visible until the second trimester morphology, leaving less time for genetic testing, especially in the time window for termination of pregnancy. Additionally, in this cohort, ultrasonographic findings did not appear to be lethal in nature. Furthermore, in eight families (29%) CHH was already a familiar diagnosis and the parents chose not to have further genetic testing during pregnancy regardless of abnormal growth.

Due to the large number of skeletal dysplasias with overlapping fetal phenotypes, NGS-based methods such as comprehensive gene panels and exome sequencing have become the methods of choice in the prenatal diagnostics. However, such methods were not yet available in the earlier years of our cohort and prenatal testing options were restricted to a handful of common variants for the most severe disease types such as diastrophic dysplasia and thanatophoric dysplasia. CHH was therefore mostly tested for postnatally.

### Delivery

**Fig. 1:**
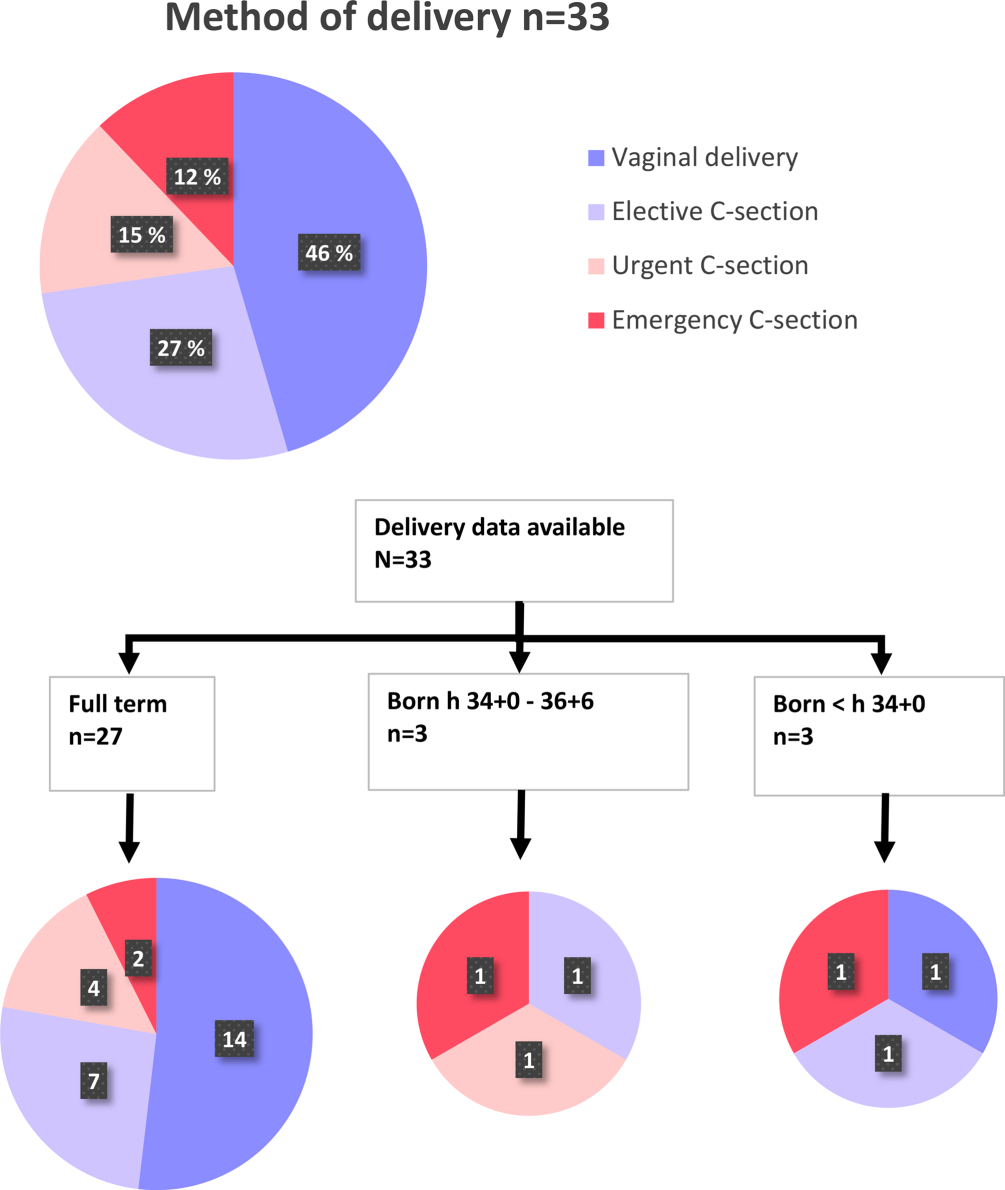
The method of delivery is depicted for all children with CHH and grouped by gestational week at the time of delivery

Delivery data were available for 33 cases. Most of the children (82%) were born at term, three (9%) were born before 34 weeks of gestation and three (9%) between 34^+0^ and 36^+6^ weeks of gestation. The method of delivery was reported for 33 patients. Less than half (46%) were born by vaginal delivery. The others were born by elective C-section (CS) (9/33; 27%), urgent CS (5/33; 15%), or emergency CS (4/33; 12%). The method of delivery is presented in Fig. 1.

CS indications differed between the urgency of the CS. In elective CS the indications included breech position (*N* = 5), oligohydramnios (*N* = 1), polyhydramnios (*N* = 1), a prenatally observed fetal bigeminy (*N* = 1) and large head combined with short long bones (*N* = 1). Urgent CS was performed because of breech position (*N* = 2), failure to progress (*N* = 2) and changes in the fetal heart rate (*N* = 1). In emergency CS the indications consisted of breech position (*N* = 1), footling breech (*N* = 2) and umbilical cord being around the baby’s neck (*N* = 1). Altogether breech position was the CS indication in 10 cases (10/18; 56%).

### Neonatal findings

**Fig. 2:**
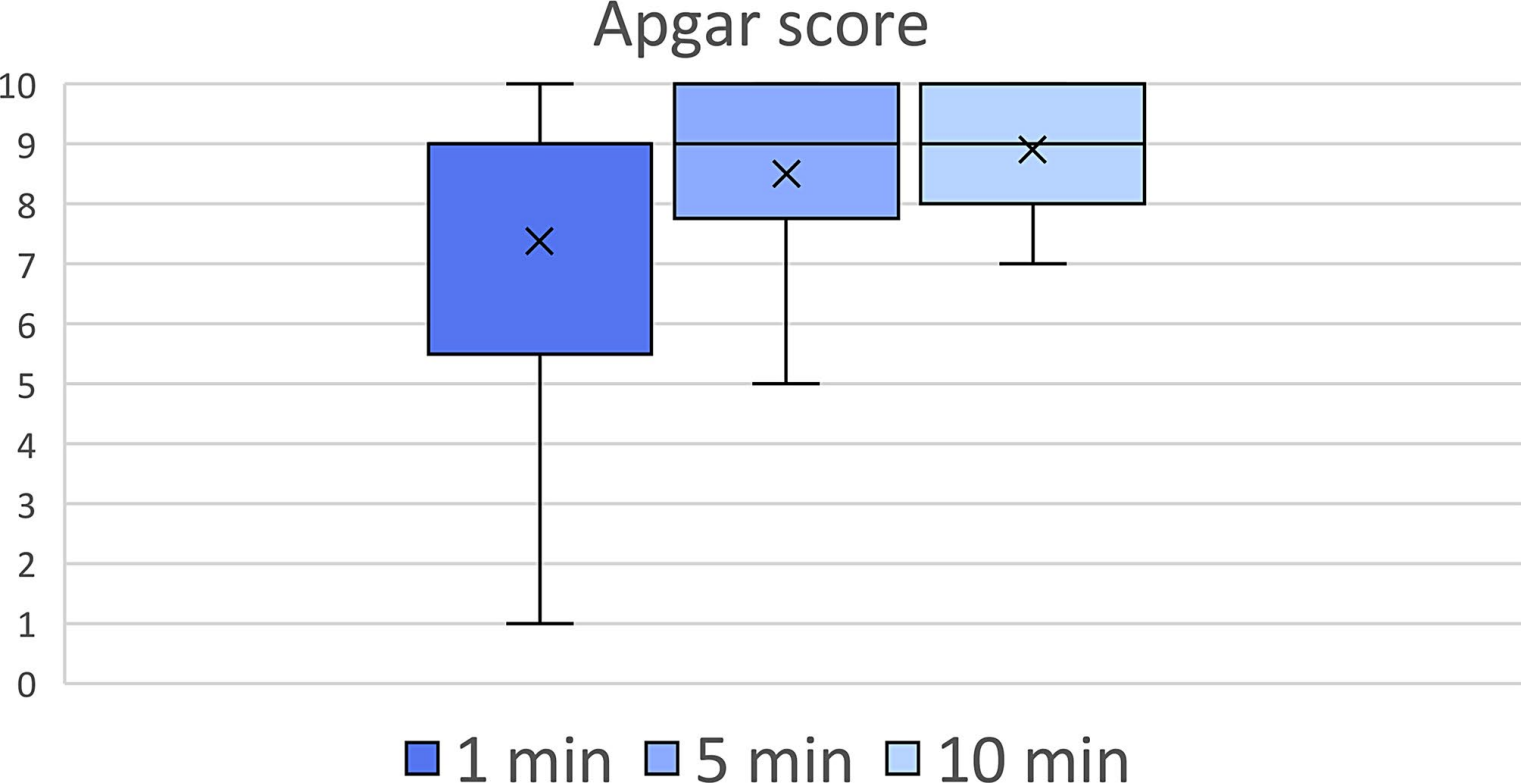
Apgar scores in the cohort of neonates with cartilage-hair hypoplasia. 1 min *n* = 21; 5 min *n* = 18; 10 min *n* = 10

Majority of the children were born in good condition but almost half of the children required respiratory support or supplementary oxygen after birth. The average Apgar score at one, five and ten minutes was 7.4 (range 1–10), 8.5 (range 5–10) and 8.9 (range 7–10) respectively. The Apgar scores are presented in Fig. 2. The median umbilical artery pH was 7.3 (range 6.70–7.36). Additional information about neonatal state was available in 19 cases. Seven (37%) children needed respiratory support, including intubation and continuous positive airway pressure (CPAP), in the immediate newborn period. Three (43%) of these children were born before 34 weeks of gestation and one (14%) was born between 34^+0^ and 36^+6^ weeks of gestation. Indications for respiratory support included hypoventilation and pulmonary hypertension. One child received resuscitation after birth. Supplementary oxygen was given through nasal cannula for 2/19 (11%) additional children.

The longest period of respiratory support continued up to four months of age. In this child the need for respiratory support began shortly after premature birth at 31^+6^ weeks of gestation because of the development of pulmonary hypertension. The neonate received respirator treatment for a week, followed by nasal CPAP treatment for another week. Supplementary oxygen was administered through a nasal cannula until the age of four months. Icterus and subsequent phototherapy was reported in one case. Low blood sugar levels were not reported.

**Fig. 3:**
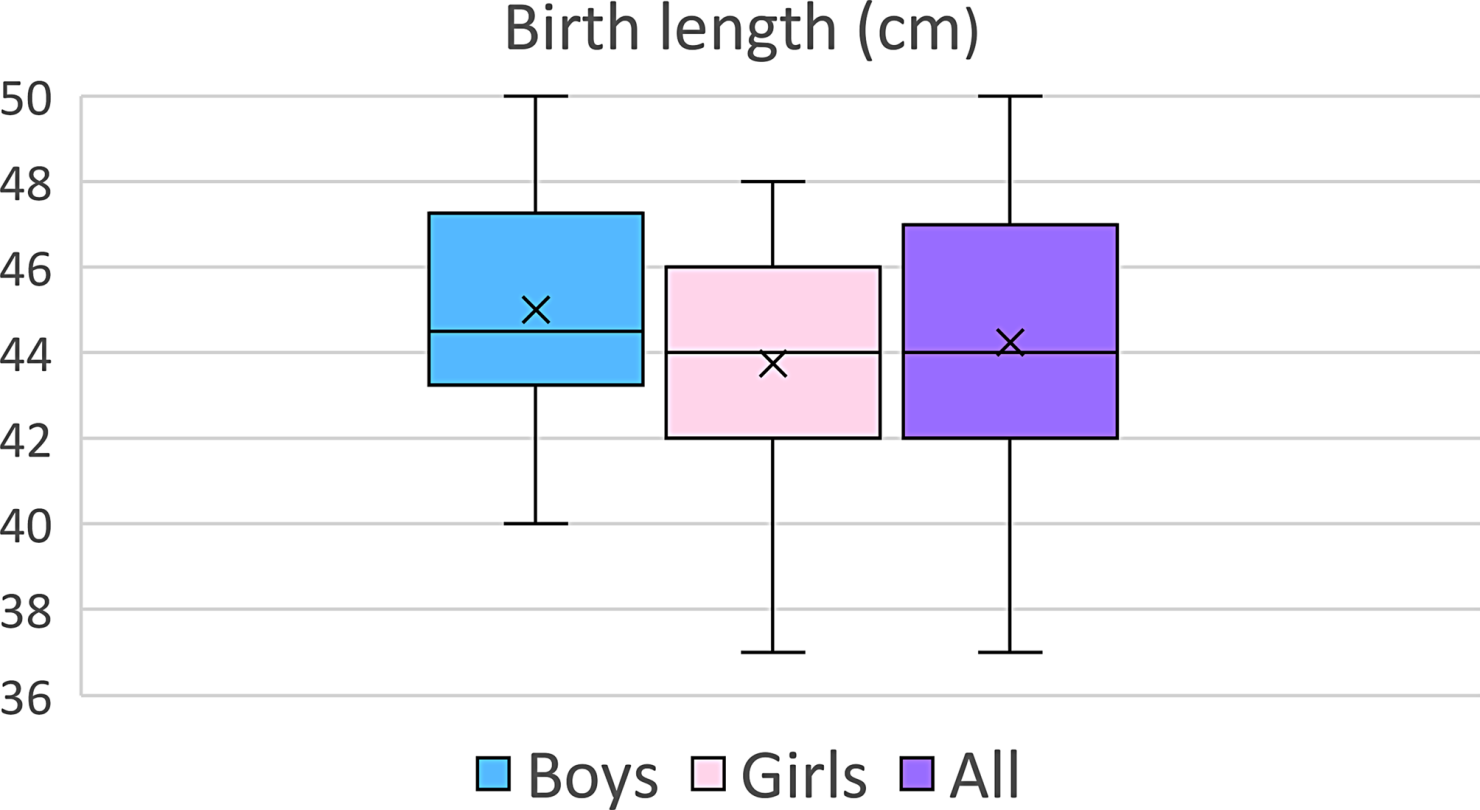
Birth lengths in centimeters for full-term (>38 gestational weeks) neonates with cartilage- hair hypoplasia. Including 10 boys and 16 girls

Birth lengths were available for 32 children. The median length for those born at term was 44.5 cm (range 40.0–50.0 cm) for boys (*N* = 10) and 44.0 cm (37.0–48.0 cm) for girls (*N* = 16) with corresponding Z-scores (corrected for gestational age) of −3.9 (range −6.2– −1.0) for boys and −3.8 (−7.3– −1.3) for girls. For two boys and two girls born at term the birth lengths were within normal variation (range −1.0 SD– −2.0 SD). For those born before 34 weeks of pregnancy (*n* = 3) the mean Z-score for birth length corrected for gestational age [27] was −4.9 (range −1.7– −7.5) and for those born between 34 + 0 and 36 + 6 weeks (*n* = 3) −4.9 (range −2.9– −7.0). In the whole cohort 27/32 (84%) had a Z-score below −2.0. The birth lengths for full-term neonates are presented in Fig. 3.

Comorbidities associated with CHH were diagnosed in the cohort during the first year of life. The incidences of HD, immunodeficiency and anemia in the cohort have been reported in a previous study [11]. Date of discharge from hospital after birth was available for 13 cases, median being 8 days and ranging from 2 days to over 6 months. The primary reasons for prolonged hospital stay included complications due to HD, anemia, immunodeficiency and/or infections. The need for red blood cell infusions ranged from one-time infusions to recurrent infusions every two weeks. Three (9%) children were diagnosed with inguinal hernia, two of which were bilateral. In the cases of bilateral inguinal hernia, the birthweights were 1490 g and 2540 g, and for the unilateral inguinal hernia 2950 g.

## Discussion

In this first study on perinatal aspects of cartilage-hair hypoplasia (CHH) we characterized the prenatal findings and the course of pregnancies and deliveries in newborns with CHH. Similar to previous studies, the Finnish founder variant in *RMRP*, n.72G > A, accounted for the majority of the cases also in this cohort [2, 11]. All parents were clinically healthy but in almost one-third of cases there was a positive family history for CHH with affected siblings or cousins, in accordance with autosomal recessive inheritance. Ultrasonographic findings were present in almost all of the cases prenatally, yet few genetic diagnoses were obtained before birth. Most pregnancies in the cohort were carried to full term, but less than half of the children were born by vaginal delivery. The children in the cohort were born shorter than average, but in relatively good health, with some requiring respiratory support.

In this cohort, ultrasound remained the primary method prompting suspicion of skeletal dysplasia, which is in keeping with literature [21, 30–34]. In general, prenatal skeletal dysplasias are visualized in ultrasonographic examinations in the second or third trimester, but cases with detection as early as before 14 weeks of gestation have been reported [30, 33, 34]. This pattern was also seen in our cohort. It is notable that ultrasound examinations didn’t show curving of long bones.

The small number of prenatal genetic diagnoses can be attributed to a few reasons. In many families of the cohort there were older siblings with CHH at the time of prenatal suspicion and many families wished for no additional testing during pregnancy. The fact that the ultrasonographic findings did not appear to be lethal contributed to many families refraining from invasive prenatal testing. In our study, there were no reports of markers associated with elevated risk of lethality such as increased NT, which has been detected in other skeletal dysplasias, for instance in severe cases of osteogenesis imperfecta (OI), leading to prenatal diagnosis [35–37].

A surprising fact in our cohort was that the proportion of CSs was more than twice as high as in the general population. In Finland 20.3% of all deliveries were CSs with planned CSs accounting for 8.3%, urgent CSs 11% and emergency CSs 0.9% in 2023 [38]. This emphasizes the disproportionately high share of all CSs (54%) and of emergency CSs (17%) in pregnancies with fetal CHH. There is little research available on the mode of delivery in fetal skeletal dysplasia. In 2018, best practice guidelines were created by a panel of multidisciplinary international experts [22]. They state that instrumental vaginal delivery should be avoided in the case of fetal skeletal dysplasia when possible due to cervical spine concerns and increased risk of intracranial hemorrhage [22, 39]. With regards to the nature of CHH, these are not the most probable complications. Furthermore, the incidence of fractures has not been reported to decrease in children with OI delivered via CS compared to vaginal delivery [40].

As CSs are associated with altered immune development, unnecessary CSs should be avoided to alleviate the risk of aggravating the disturbed immunity in children with CHH [41]. On the other hand, the number of breech positions and other malpresentations was higher than expected – in total more than 30% – and led to urgent and emergency CSs in many of the cases. Breech presentation affects 3–4% of births in the general population and has been reported in pregnancies with fetal skeletal dysplasia [42–44]. Growth restriction, and congenital anomalies, as well as preterm birth, are known risk factors for breech position, which offer possible explanations for the increased incidence in this cohort [44]. Based on our findings, CHH alone should not be seen as an indication for a CS but the elevated risk for malpresentation in CHH should be taken into account in the planning and management of delivery.

All the children in the cohort were born shorter than average with only a few children having a birth length above −2.0 SD. Cohorts with different genetic backgrounds have been reported to have birth lengths within normal range and it is probable that the FFV is responsible for the more severe phenotype in our cohort [25]. Based on previous research, the importance of birth length should be emphasized as a possible prognostic factor of the clinical course in CHH [11, 45]. In addition to short birth length, some children in the cohort presented with an inguinal hernia in the first year of life. Known risk factors for inguinal hernias include low birth weight and prematurity and these factors may explain the relatively high incidence in this cohort [46–48].

In skeletal dysplasias respiratory problems in infancy are caused by multiple etiologies including thoracic and craniofacial abnormalities [49, 50]. In CHH, immunodeficiency may further contribute to respiratory challenges [4, 9]. In the general population the incidence of respiratory distress in the neonatal period has been reported to be 7% [51]. Partly the need for respiratory support in the cohort can be attributed to prematurity but based on our results even in full-term CHH neonates respiratory support is frequently needed and should be appropriately managed. The association of CHH and other comorbidities including HD, anemia and lymphopenia have been recognized for quite some time [5–8, 10–14]. The prevalence of HD among Finnish CHH patients has been reported to be 7–25% [11, 12]. Early suspicion and detection of HD is of utmost importance in the neonatal care of CHH patients.

Although this is the most extensive neonatal cohort of subjects with CHH to date, it has its limitations due to the rarity of CHH. The fact that the children in the cohort were born in different hospitals across Finland limited the availability of the data to some extent. Furthermore, reports of prenatal findings in the pediatric hospital records were in some cases incomplete. Because of autosomal recessive inheritance, the mothers are carriers of an *RMRP* variant. However, we did not have genetic data for the mothers. We have previously shown that parents of CHH children have normal height [1, 3] and do not have an increased risk of cancer [6, 17, 18]. Therefore, it is unlikely that the mothers’ carrier status would have an impact on the neonatal outcome. Despite these limitations we feel that our findings in the unique cohort provide valuable new information on perinatal aspects in CHH.

In conclusion, our study of perinatal outcomes in a cohort of 35 Finnish infants with CHH indicated that the growth failure is usually detected prenatally. Nevertheless, only a minority of the cases were genetically diagnosed prenatally. Most of the pregnancies were carried to full term but less than half of the children were born by normal vaginal delivery leading to the proportion of elective, urgent and emergency CSs being higher than expected with malpresentations being the main indication. Although the children were born in relatively good health, the median birth length was significantly below normal mean for both sexes. High prevalence of HD and of anemia requiring transfusions influenced neonatal patient management. More research is needed to further examine the early findings and diagnostic processes in CHH, to optimize management of pregnancies and deliveries, and to determine the potential long-term effects of CS in these immunocompromised patients.

## Data Availability

The data that support the findings of this study are available from the corresponding author upon reasonable request. The data are not publicly available due to privacy or ethical restrictions.
